# Supplementary material for: A Prospective, Case-Control Study of Serum Metabolomics in Neonates with Late-Onset Sepsis and Necrotizing Enterocolitis
Source: J Clin Med. 2022 Sep 7;11(18):5270. doi: 10.3390/jcm11185270 (PMC9505627; doi:10.3390/jcm11185270)
Supplement: Supplementary file 1 [file jcm-11-05270-s001.zip › Supplementary material Tables S1 and S2.pdf]

Supplementary Table S1. Demographic, perinatal and clinical – laboratory characteristics of LOS and control group.

Descriptive variable	Septic neonates: All cases (n = 15)	Septic neonates: Confirmed LOS (n=9)	Septic neonates: Possible LOS (n=6)	Controls (n=17)	P value*
**Demographics-perinatal characteristics**					
Gestational age (weeks)	33.7±3.73	34.4±3.61	32.7±3.98	34.1±3.4	0.79
Birth weight (g)	2204±899	2256±936	2127±921	2170±904	0.91
Male sex	7 (46.7)	3 (33.3)	4 (66.6)	13(76.5)	0.14
Prematurity	12 (83.3)	5 (83.3)	5 (83.3)	13 (81.3)	1
Apgar score					
1st min	6.8±1.7	6.56±2.13	7.17±0.75	7.6±1	0.12
5th min	8.3±0.6	8.22±0.67	8.67±0.52	8.8±0.66	0.09
Caesarian section	11 (73.3)	7 (77.8)	4 (66.7)	11 (64.7)	0.71
Prenatal steroid	9 (64.3)	4 (45.6)	5 (83.3)	10 (58.8)	0.76
Small for gestational age	0 (0)	0 (0)	0 (0)	2 (12.5)	0.49
Premature rupture of membranes > 18 hrs	1 (6.7)	0 (0)	1 (16.7)	2 (12.5)	1
Time (postnatal day) of 1st blood sampling	11±11.7	6±1.00	16.8±15.9	11.8±10.8	0.62
**Clinical characteristics (day 0)**					
Invasive mechanical ventilation	9 (60)	5 (55.5)	4 (66.6)	3 (17.6)	0.07
Inotropes	7 (46.6)	5 (62.5)	2 (33.3)	0 (0)	**0.001**
Antibiotics	12 (75)	6 (50)	6 (50)	0 (0)	**<0.001**
Full enteral feeding	1 (7.1)	0	1 (16.7)	10 (58.8)	**<0.001**
Total parenteral nutrition	6 (42.9)	2 (25)	4 (66.7)	0	**<0.001**
Partial enteral feeding	7 (50)	6 (75)	1 (16.7)	7 (41.2)	**<0.001**
**Laboratory findings**					
C-reactive protein (mg/dL)					
Day 0	46.7±35.7	53.1±44.4	38.2±20.2	4.36±3.84	**<0.001**
Day 1	96.2±4.89	104±63.3	82.9±53.5	4.89±3.41	**0.003**
White blood count (K/μL) - Day 0	13747±9597	14468±10351	12787±9355	10556± 2545	0.32
Immature /total neutrophils > 0.2 – Day 0	6	4	2	0	
Platelet count (K/μL) - Day 0	130593 ±120916	116913 ±130357	148833 ±116295	350429 ±125222	**<0.001**
Blood lactate (mg/dL) - Day 0	20.2±9.00	23.3±10.8	16.2±3.60	13.9±5.15	0.023
Blood glucose (mg/dL) - Day 0	121±44.1	129±47.6	111±41.0	98.1±21.2	0.22
Serum creatinine (mg/dL)					
Day 0	0.67±0.17	0.7±0.17	0.64±0.2	0.63±0.15	0.50
Day 3	0.66±0.18	0.67±0.19	0.65±0.18	0.64±0.08	0.70

Quantitative variables are shown as mean ± SD and qualitative variables as numbers and % percentage within parenthesis.

* p value is referred to the comparison between sepsis and control groups.

Table S2. Demographic, perinatal and clinical – laboratory characteristics of NEC and control group.

Descriptive variable	Neonates with NEC			Controls (n=18)	p value*
	All cases (n = 19)	Confirmed NEC (n=13)	Suspected NEC (n=6)		
**Demographics-perinatal characteristics**					
Gestational age (weeks)	33.6±2.17	33.5±2.44	33.8±1.6	34.1±3.31	0.56
Birth weight (g)	1992±550	2002±595	1968±490	2208±892	0.38
Male sex	12 (63.2)	7 (53.8)	5 (83.3)	14(77.8)	0.48
Prematurity					
Apgar score	7.2±1.3	6.8±1.4	8±0	7.6±1.0	0.24
1st min	8.4±0.8	8.2±0.9	8.8±0.4	8.8±0.6	0.11
5th min	13(68.4)	8 (61.5)	5 (83.3)	12 (67.7)	0.91
Caesarian section	11 (57.9)	8 (61.5)	3 (50)	10(55.6)	0.89
Prenatal steroid	6(31.6)	3(23.1)	3(50)	2 (10.5)	0.13
Small for gestational age	5 (26.3)	4 (30.8)	0 (0.0)	2(11.8)	0.4
Premature rupture of membranes > 18 hrs	9.81±5.13	11±5.26	7.25 ±4.12	11.7±10.5	0.49
**Clinical characteristics (day 0)**					
Invasive mechanical ventilation	3(15.8)	3 (23.1)	0	3(16.7)	1
Inotropes	0	0	0	0	
Full enteral feeding	2 (11.1)	1 (7.7)	1(20)	11 (61.1)	<0.001
Total parenteral nutrition	12 (66.7)	9 (69.2)	3 (60)	0	<0.001
Partial enteral feeding	4 (22.2)	3 (23.1)	1 (20)	7 (38.9)	<0.001
**Laboratory findings**					
C-reactive protein (mg/dL)					
Day 0	21±30.7	23±34.5	15.8±19.8	4.36±3.68	0.13
Day 1	36.3±88.1	52.3±116	13.8±3.19	4.89±3.41	0.38
White blood count (K/μL) - Day 0	11054±7499	12560±8401	7790±3799	10552±2445	0.81
Platelet count (K/μL) - Day 0	313579 ±138452	330462 ±135547	277000 ±150221	354000 ±121457	0.37
Blood lactate (mg/dL) - Day 0	13.7±5.65	13.5±6.64	14.3±3.33	14.4±5.36	0.76
Blood glucose (mg/dL) - Day 0	104.2±37.8	105.5±44.8	101.3±17.6	98.5±20.6	0.58

Quantitative variables are shown as mean ± SD and qualitative variables as numbers and % percentage within parenthesis.

* p value is referred to the comparison between all NEC cases and control groups.
