# Supplementary material for: A Prospective, Case-Control Study of Serum Metabolomics in Neonates with Late-Onset Sepsis and Necrotizing Enterocolitis
Source: J Clin Med. 2022 Sep 7;11(18):5270. doi: 10.3390/jcm11185270 (PMC9505627; doi:10.3390/jcm11185270)
Supplement: Supplementary file 1 [file jcm-11-05270-s001.zip › Supplementary material-File S1.pdf]

The modified Bell's staging criteria

Stage 1 (suspected NEC) is characterized by nonspecific radiologic signs (temperature instability, apnea and bradycardia, lethargy, mild abdominal distension).

Stage 2 (proven NEC) is characterized by the radiographic findings of pneumatosis intestinalis and portal venous gas, and mild metabolic acidosis and ileus.

Stage 3 (advanced NEC) is characterized by the finding of pneumatosis intestinalis plus significant systemic signs (eg, severe metabolic acidosis and disseminated intravascular coagulation [DIC]) and subsequent perforation. Moreover, cases of NEC were categorized as medical NEC (improvement without surgery) or surgical NEC (underwent laparotomy or/and peritoneal drainage).

Reference

Kliegman RM, Walsh MC. Neonatal necrotizing enterocolitis: pathogenesis, classification, and spectrum of illness. Curr Probl Pediatr. 1987;17(4):213-288.
